# Microbial Metabolites in Multiple Sclerosis: Implications for Pathogenesis and Treatment

**DOI:** 10.3389/fnins.2022.885031

**Published:** 2022-04-28

**Authors:** Eduardo Duarte-Silva, Sven G. Meuth, Christina Alves Peixoto

**Affiliations:** ^1^Laboratory of Ultrastructure, Aggeu Magalhães Institute (IAM), Recife, Brazil; ^2^Postgraduate Program in Biosciences and Biotechnology for Health (PPGBBS), Oswaldo Cruz Foundation (FIOCRUZ-PE)/Aggeu Magalhães Institute (IAM), Recife, Brazil; ^3^Network of Immunity in Infection, Malignancy and Autoimmunity (NIIMA), Universal Scientific Education and Research Network (USERN), Recife, Brazil; ^4^Department of Neurology, Medical Faculty, University Hospital Düsseldorf, Düsseldorf, Germany; ^5^National Institute of Science and Technology on Neuroimmunomodulation (INCT-NIM), Oswaldo Cruz Institute, Oswaldo Cruz Foundation, Rio de Janeiro, Brazil

**Keywords:** experimental autoimmune encephalomyelitis (EAE), multiple sclerosis (MS), short-chain fatty acids, urolithins, polyamines, gut microbiota-derived metabolites

## Abstract

Metabolites produced by the gut microbiota have been shown to play an important role in numerous inflammatory, neuropsychiatric, and neurodegenerative diseases. Specifically, microbial metabolites have been implicated in the modulation of innate and adaptive immunity, especially in the generation of regulatory T cells (Tregs), which are key regulators of multiple sclerosis (MS) pathogenesis. Furthermore, they affect processes relevant to MS pathophysiology, such as inflammation and demyelination, which makes them attractive molecules to be explored as therapeutics in MS. In this review, we discuss the importance of these metabolites as factors contributing to disease pathogenesis and as therapeutic targets in MS. Establishing an improved understanding of these gut-microbiota derived metabolites may provide new avenues for the treatment of MS.

## Introduction

Multiple Sclerosis (MS) is a chronic inflammatory disease that affects the central nervous system (CNS), causing demyelination and degeneration of neurons and axons, consequently causing neurological disability in affected patients (Baecher-Allan et al., 2018). Currently, immunomodulatory drugs or antibodies are a critical part of the mainstay of treatment, but have significant side effects, namely immune suppression (Baecher-Allan et al., 2018). Therefore, the discovery of new therapeutics that are both effective and not immunosuppressive is a key challenge in MS research. In this regard, the role of the metabolites produced by the gut microbiota is of increasing interest. It is known that some metabolites, such as p-cresol sulfate, indoxyl sulfate, and n-phenylacetylglutamine have neurotoxic effects (Ntranos et al., 2021), while others have a clear role in normal host physiology, for example, immune modulation, tolerance (van der Hee and Wells, 2021), and in the gut-brain axis (Dalile et al., 2019; O’Riordan et al., 2022). Therapies based on the modulation of gut microbiota or even personalized nutrition are now being explored and may be considered promising therapeutic approaches when used either as a monotherapy or in combination with standard treatment. Although there are already some published reviews on the current topic (Haase et al., 2020; Fettig and Osborne, 2021), here we discuss the more recent findings and the role of other metabolites not covered by previous reviews. Therefore, the aim of this review is to describe the role of microbially derived molecules in the pathogenesis and treatment of MS, focusing on short-chain fatty acids (SCFAs), polyamines, and urolithins.

## The Role of the Gut Microbiota in the Pathogenesis of Multiple Sclerosis

The gut microbiota refers to a community of microorganisms – bacteria, viruses, eukaryotes, and archaea – that inhabit the intestine of the host and have undergone co-evolution over thousands of years. While co-evolving, these microorganisms have utilized diverse mechanisms to ensure their own survival, while providing benefits to the host, such as the digestion of complex carbohydrates (Rinninella et al., 2019). Although the bacteria species are highly diverse, they are often clustered into the two following main phyla: Bacteroidetes and Firmicutes (Rinninella et al., 2019).

In recent decades, there has been a rise in the number of studies addressing the role of the gut microbiota and their metabolites in inflammatory, neuropsychiatric and neurodegenerative/neuroinflammatory diseases, such as inflammatory bowel disease (IBD), major depressive disorder (MDD), and MS. To date, a large quantity of data demonstrating the involvement of the gut microbiota in these aforementioned diseases has accumulated (Wekerle, 2017; Cruz-Pereira et al., 2020; Kadowaki and Quintana, 2020). This has allowed for the development of gut microbiota-targeted therapies, such as probiotics, psychobiotics (Dinan et al., 2013), prebiotics (Paiva et al., 2020), fecal microbiota transplant (FMT) (Kelly et al., 2016), and personalized nutrition-based interventions (Duarte-Silva et al., 2021), as well as for a deeper understanding of disease pathophysiology. Further to this, changes in the composition of the gut microbiota have been causally associated with the development of experimental autoimmune encephalomyelitis (EAE), a mouse model of MS (Berer et al., 2017) and MS patients harbor a different gut microbiome composition when compared to healthy individuals (Miyake et al., 2015; Chen et al., 2016). Specifically, high numbers of *Methanobrevibacter* (Euryarchaeota phylum) and *Akkermansia* (Verrucomicrobia phylum) were reported in relapsing-remitting MS (RRMS) patients, with reduced numbers of *Butyricimonas* and *Prevotella* (both belonging to Bacteroidetes phylum). Of note, untreated patients had reduced abundance of *Collinsella* and *Slackia* (Coriobacteriaceae family) and *Prevotella* and patients on disease-modifying therapy had higher abundance of *Sutterella* and *Prevotella*, and lower numbers of *Sarcina* (Jangi et al., 2016). Interestingly, the abundance of *Methanobrevibacter* and *Akkermansia* was positively associated with the induction of innate and adaptive immunity gene pathways in T cells and monocytes, while *Butyricimonas*, known to produce butyrate, negatively correlated with these pro-inflammatory genes (Jangi et al., 2016). This suggests these microbes play either a role in, or change consequential to, disease pathogenesis. In addition to RRMS, gut microbiota changes were recently characterized in progressive MS (Kozhieva et al., 2019; Reynders et al., 2020; Cox et al., 2021). Among the most significant findings was that progressive MS patients had higher abundance of *Ruthenibacterium lactatiformans*, *Akkermansia*, *Enterobacteriaceae*, *Bifidobacterium animalis*, *Dorea massiliensis, Clostridium g24 FCEY*, and *Ruminococcaceae FJ366134*, and reduced abundance of *Lachnospiraceae PAC001046* and *Phascolarctobacterium faecium* (Cox et al., 2021). More recently, a key role of gut IL-17 was established as a underpinning mechanism triggering the development of EAE (Regen et al., 2021), which corroborates the key role of the gut microbiota in CNS autoimmunity initiation.

## Bacterial Metabolites

The metabolites produced by the gut microbiota can be classified overall into three categories (Yang and Cong, 2021): (1) diet-derived, but processed by gut microbiota, (2) host-derived, but processed by the gut microbiota, and (3) produced directly by the gut microbiota (*de novo* synthesis). These metabolites, comprising, for instance, SCFAs and tryptophan metabolites, secondary bile acids (2BAs) and polyamines, exert a variety of metabolic and immune effects (Kim, 2018; Yang and Cong, 2021), thus contributing to host homeostasis. However, the dysfunction in the production of these molecules is, as expected, implicated in plethora of metabolic, autoimmune, neurodegenerative, and neuropsychiatric disorders, such as MS and MDD (Caspani et al., 2019; Merchak and Gaultier, 2020). This highlights the need for a more in-depth understanding of the role of these molecules in health and disease, as this may also aid in the establishment of novel therapeutics, particularly given that some of these metabolites are widely implicated in the modulation of Th17 lymphocytes or in the induction of regulatory T cells (Tregs) and tolerogenic dendritic cells (DCs) (Kim, 2018). In the following sections, we describe the role of SCFAS, polyamines and urolithins in the pathogenesis of MS and describe how these metabolites may be utilized for novel treatments. For an updated review on tryptophan metabolites and 2BAs in MS, the reader is referred to another recently published review paper (Fettig and Osborne, 2021).

## Short-Chain Fatty Acids

Short-chain fatty acids are by-products of the fermentation of complex and indigestible carbohydrates in the colon and comprise, for instance, acetate (C2), propionate (C3), and butyrate (C4), which are known to be potent immune modulators (Corrêa-Oliveira et al., 2016). In general, the source of SCFAs is dietary fiber, but they can also be generated in lower quantities from proteins and peptides (Kim, 2021). SCFAs can act in a receptor-dependent and receptor-independent fashion and affect the host physiology both locally and systemically when in the bloodstream. The most studied SCFAs receptors are GPR41 (FFAR3), GPR43 (FFAR2), GPR109a/hydroxycarboxylic acid receptor (HCA2R), and Olfr78, and binding to these receptors usually triggers the activation of complex intracellular signaling pathways, such as the ERK1/2 pathway, which modulate different cellular functions, such as activation and differentiation. SCFAs can diffuse into the cells or enter *via* transporters located in the apical and/or basolateral cell membrane. After entering the cell, SCFAs can inhibit histone deacetylases (HDACs) and favor protein acetylation, which directly influences gene expression (Kim, 2021). Signaling by SCFAs has been shown to modulate the activity of innate and adaptive immune cells (Kim, 2021; Yang and Cong, 2021) and to be modulated by health status and diet (Kim, 2021), ultimately affecting host homeostasis. Of note, some microbes that produce SCFAs have been characterized, such as *Faecalibacterium prausnitzii* (Deleu et al., 2021), *Akkermansia muciniphila*, and *Roseburia inulinivorans* (Kim, 2018) and *Butyricimonas* (Jangi et al., 2016).

The relevant role of SCFAs in MS pathophysiology is suggested by MS patients having reduced levels of these molecules (Saresella et al., 2020; Olsson et al., 2021; Trend et al., 2021). Notably, long-term secondary progressive (SPMS) patients were shown to have reduced blood levels of acetate, propionate, and butyrate in comparison to healthy individuals (Park et al., 2019). Identical findings were also reported in the feces of RRMS patients (Takewaki et al., 2020), suggesting that these changes occur regardless of disease type, although timing seems to be an important factor driving these changes. Specifically, only decreased levels of propionate were detected in the serum of clinically isolated syndrome (CIS) patients (Trend et al., 2021) and only acetate (Olsson et al., 2021) and propionate (Duscha et al., 2020) were found to be reduced in newly diagnosed MS patients. Moreover, administration of SCFAs, notably acetate, improved disease severity in EAE in a IL-10-dependent fashion (Park et al., 2019). Similar findings were also reported after propionate administration in EAE mice (Haghikia et al., 2015). Furthermore, reduced CNS inflammation and demyelination were observed after preventive treatment with butyrate (Calvo-Barreiro et al., 2021) and propionate (Haghikia et al., 2015) in EAE mice. Intriguingly, butyrate administered after disease onset had little impact on disease course (Calvo-Barreiro et al., 2021), while propionate administered in the same fashion resulted in recovery of axonal density, even though in the preventive approach butyrate reduced demyelination and immune cell infiltration. These results suggest that restoring SCFAs levels may be a promising therapeutic approach to treat MS, especially *via* a diet rich in fiber (Mizuno et al., 2017); however, the outcomes may depend on the adopted therapeutic regimen. This raises the possibility that supplementation with propionate may help in MS prevention or at least allow for a milder course in comparison with a diet deficient in SCFAs. In fact, this is corroborated by a subsequent study on propionate supplementation in a cohort of MS patients (Duscha et al., 2020). Propionic acid was shown to improve MS symptoms *via* increasing the number of peripheral Tregs and their suppressive capacity *ex vivo* and *in vitro* in a IL-10 dependent manner (Duscha et al., 2020).

In addition to the generation and modulation of peripheral Tregs, a role for intestinal Treg in the suppression of CNS autoimmunity has also been demonstrated. Propionate increased Treg numbers in the small intestine and increased gene expression of intestinal IL-10, TGF-β, and Foxp3 (Haghikia et al., 2015). Aside from direct effects on Tregs, propionate also triggers Treg induction *via* gut microbiota-dependent mechanisms. Gut microbiota of MS patients treated with propionate triggered increased expression of genes involved with Treg differentiation in an intestinal organ culture system (Duscha et al., 2020). Moreover, transfer of propionate-treated Tregs to EAE mice reduced disease severity (Haghikia et al., 2015). Finally, propionate also increases Treg number in the spinal cord and spleen (Haase et al., 2021). These data show that propionate improves CNS autoimmunity by increasing the frequency of Treg not only in the periphery, but also in the CNS, where they likely counteract the ongoing inflammation. Of note, IL-17A in the gut is sufficient to restore the susceptibility of mice to develop EAE because of its modulatory effects on the gut microbiota. In fact, IL-17A expression in intestinal epithelial cells *per se* was shown to reduce 13 operational taxonomical units (OTUs) that were more abundant in mice with lower susceptibility to EAE, notably OTU 0754 (Clostridiales_uc) (Regen et al., 2021). However, the precise bacterial species driving increased susceptibility to EAE, their metabolites and *modus operandi* are yet to be determined. However, it is worth mentioning that, in general, some species of *Clostridiales* are SCFAs producers (Deleu et al., 2021). It is therefore reasonable to hypothesize that higher abundance of SCFAs-producers may be a key factor influencing the susceptibility to autoimmunity, which is markedly influenced by Tregs. Furthermore, accumulation of Tregs in the gut caused by SCFAs may favor a more tolerogenic microenvironment, thus inhibiting autoimmunity.

In addition to these changes of the most common SCFAs, research has also shed light on the role of valerate/pentanoate (C5) in CNS autoimmunity. Through epigenetic and metabolic modulation, pentanoate was shown to inhibit immune cell infiltration into the brain and intestine, particularly Th17 lymphocytes, and to inhibit IL-17A secretion and the expression of RORγt and STAT3 (Luu et al., 2019). Furthermore, it also ameliorated EAE by increasing the secretion of IL-10 by regulatory B cells (Bregs), which also reduced the number of effector T cells (Teffs) in the gut (Luu et al., 2019). In contrast, SCFAs administration increased the numbers of IL-17^+^ cells in the CNS and draining lymphatic system (Park et al., 2019), as well as IL-17 production (Mizuno et al., 2017). Notably, acetate-treated Th17 lymphocytes caused a more severe EAE course (Park et al., 2019). These results suggest that autoimmunity can occur not only due to an overall reduction of SCFAs, but also due to imbalance in their respective quantities, resulting in increased/decreased levels of one type of SCFAs in comparison to the other types. Consequently, this could skew the immune response toward a Th17 response. Surprisingly, increased levels of acetate were detected in the plasma of MS patients and were associated with greater neurological disability and higher number of CD8^+^ IL-17^+^ T cells (Pérez-Pérez et al., 2020). However, the context and nature of the disease under investigation must also be taken into consideration, as SCFAs *per se* can have detrimental effects and worsen antibody-mediated diseases (Mizuno et al., 2017).

Apart from SCFAs, it is important to mention that MS patients were shown to have increased levels of caproic acid (C6) (Saresella et al., 2020), a medium-chain fatty acid (MCFA) previously shown to induce the differentiation of Th17 and Th1 cells and to decrease the differentiation of Tregs (Haghikia et al., 2015). Since this compound is mainly obtained through dietary intake, this highlights the key role of environmental factors as triggers of autoimmunity. In line with this, a study found that long-chain fatty acids (LCFAs) commonly present in Western diet, such as lauric acid, and indeed a diet rich in this compound alone given experimentally increased EAE severity by augmenting the differentiation and migration of Th17 cells to the spinal cord and lamina propria of the small intestine, while SCFAs, notably propionate, exerted beneficial effects by promoting Treg differentiation and proliferation in the small intestine, which counteracted the effects of a lauric acid-rich diet. Of note, this diet reduced the levels of all SCFAs, while increasing the levels of MCFAs, notably caproic acid (Haghikia et al., 2015). Similarly, reduced propionate levels were detected in the feces of obese MS patients in comparison to those that are non-obese (Haase et al., 2021). Of note, increased BMI is associated with faster progression to MS and more aggressive disease (Manuel Escobar et al., 2022). As propionic acid shifts the Th cells toward a Treg profile, these patients also had reduced numbers of Tregs in the blood, while the frequency of Th17 cells was high (Haase et al., 2021).

## Polyamines

Polyamines comprise of spermidine, spermine, and putrescine, which are natural molecules derived from the L-arginine metabolism that can be produced by the host or the some bacteria of the gut microbiota (Yang and Cong, 2021), such as *Bacteroides thetaiotaomicron* and *Fusobacterium varium* (Noack et al., 2000) and also bacteria from the genera Enterococcus and Bifidobacterium (Pugin et al., 2017). They mediate a plethora of cellular effects, such as cell growth and survival, particularly in the context of cancer (Casero et al., 2018). Furthermore, they have also been implicated in the differentiation of T helper cells (Puleston et al., 2021), notably Th17 cells (Wagner et al., 2021), which makes them attractive targets in MS and other Th17-mediated diseases.

Research has shown that polyamines can dampen neuroinflammation *via* modulation of microglia/macrophages or modulation of T cells. For instance, LPS-stimulated microglia treated with spermidine secreted less pro-inflammatory mediators, such as nitric oxide (NO), PGE_2_, IL-6, and TNF-α likely *via* inhibition of NFκB (Choi and Park, 2012). In the same vein, macrophages treated with spermidine were shown to have reduced activation of the NFκB pathway and migrated less frequently to the spinal cord in EAE (Yang et al., 2016). Interestingly, spermidine is also able to modulate antigen presentation in macrophages, as lowered levels of costimulatory molecules CD80 and CD86 were observed after the treatment with this molecule, consequently resulting in less proliferation of T cells (Yang et al., 2016). Furthermore, spermidine caused a shift to the M2 profile, increasing the levels of Arginase-1 (Arg-1), Ym1, and Rentla, consequently leading to amelioration of EAE in a manner independent of IL-10 and TGF-β. Interestingly, adoptive transfer of macrophages, and not CD4^+^ T cells, from mice treated with spermidine to EAE mice reduced disease severity, which was dependent of Arg-1 (Yang et al., 2016).

Regarding T cells, spermidine was shown to promote FoxP3^+^ Treg differentiation *in vitro* and in the small intestine and in the colon of mice, while causing a reduction in the frequency of IL-17 producing cells *in vitro* (Carriche et al., 2021). Strikingly, polyamines were shown to be essential for the induction of pathogenic Th17 (pTh17) cells, and manipulation of the enzymes in the polyamine pathway was able to alter the frequency of IL-17^+^ cells, likely *via* modulation of STAT3 and RORγt (Wagner et al., 2021). In addition, polyamine administration generated more Foxp3^+^ cells and ameliorated EAE severity (Wagner et al., 2021). Furthermore, reduced immune cell infiltration into the CNS has also been detected after spermidine treatment (Yang et al., 2016), which could be also be a result of decreased release of astrocyte-derived chemokines, such as MIP-1α, MCP-1, and RANTES, observed *in vitro* after the treatment with spermidine (Guo et al., 2011) or *via* direct inhibition of LFA-1 on T lymphocytes by spermine and spermidine (Soda et al., 2005). Finally, spermidine has also been shown to decrease astrocyte and microglia number in EAE (Guo et al., 2011). Altogether, these findings support the notion that targeting the polyamine pathway may be a promising therapeutic approach to tackle MS.

## Urolithins

Ellagitannins (ETs) and ellagic acid (EA) are polyphenolic compounds abundantly present in walnuts and fruits, such as strawberries and pomegranate (D’Amico et al., 2021). The metabolization of EA by the specific members of the gut microbiota, such as *Gordonibacter urolithinfaciens* and *Gordonibacter pamelaeae* (Eggerthellaceae family) (Selma et al., 2014) generates another class of metabolites known as urolithins, comprising urolithin A–D. Over the last few years, research has demonstrated that urolithin A (UA) has anti-inflammatory, anti-aging, and neuroprotective effects (D’Amico et al., 2021). Of note, production of UA does not occur in all individuals as it relies on the composition of the gut microbiota (D’Amico et al., 2021). Therefore, direct supplementation with UA is one possible strategy to overcome this limitation.

Although the understanding of the mechanism of action of UA in the context of neuroinflammation, neurodegeneration and autoimmunity is still in its infancy, a body of evidence supports the notion of UA being a promising therapeutic approach. Inhibition of inflammation has been observed after UA administration *in vitro* (DaSilva et al., 2019; Singh et al., 2019) and in different mice models, such as APP/PS1 mice (Gong et al., 2019) and in a model of TBNS- and DSS-induced colitis (Singh et al., 2019). Interestingly, the UA precursor EA failed to inhibit neuroinflammation in EAE, although it was effective in the prevention of loss of MBP and sphingolipids in the cortex and spinal cord, respectively (Busto et al., 2018). Of note, UA and urolithin B (UB) were able to increase the synthesis of ceramide *in vitro*, which is decreased during acute EAE (Busto et al., 2018). Furthermore, attenuation of cognitive deficits has also been achieved after the treatment with UA (Gong et al., 2019) and urolithin B (UB) (Chen et al., 2021). Altogether, these results suggest that the urolithin precursor is only able to have an effect on the consequences of inflammation (for instance, demyelination), not the cause of inflammation, as is the case for urolithins.

An important aspect of the mechanism of action of urolithins, specifically UA, is the modulation of gut barrier function, which has direct implications for MS, as studies have shown compromised intestinal epithelial barrier may play an important role in MS pathogenesis (Buscarinu et al., 2017; Camara-Lemarroy et al., 2018, 2020; Saresella et al., 2020; Pellizoni et al., 2021). However, mechanistic studies in the field are still lacking. Through the modulation of tight junction proteins, UA was shown to decrease the epithelial intestinal permeability and attenuate colitis in a Ahr-Nrf2-dependent fashion (Singh et al., 2019).

Apart from its effects on the regulation of gut barrier integrity, UA can also modulate T cells, DCs and microglia (Zhang et al., 2019; Shen et al., 2021). By interfering with the calcium machinery of T cells in a miR-10a-5p-dependent manner, UA was shown to inhibit CD4^+^ T cell activation and proliferation *in vitro* (Zhang et al., 2019). In a similar fashion, reduced Th17 differentiation and lower levels of IL-17 were detected after the treatment with UA *in vitro*. Furthermore, DCs pre-treated with UA were less stimulatory of Th17 lymphocyte differentiation (Shen et al., 2021). Interestingly, UA treatment reduced the levels of CD80, CD86, and MHC-II on DCs stimulated with LPS (Shen et al., 2021). In EAE, either preventive or therapeutic treatment with UA lowered disease severity, likely due to inhibition of M1 microglia, infiltration of monocytes, Th1 and Th17 cells in the CNS, and even MOG-specific Th17 cells (Shen et al., 2021). These results suggest that UA supplementation may hold promise as a therapeutic approach to tackle MS, but clinical trials and more mechanistic pre-clinical studies are certainly still needed.

## Implications for Behavioral Changes in Multiple Sclerosis

Multiple sclerosis is frequently accompanied by neuropsychiatric comorbidities, such as anxiety and depression and behavioral dysfunction, which is also often observed in a similar form during the pre-symptomatic phase of EAE (Duarte-Silva et al., 2019). Microbial metabolites have been extensively implicated in the pathogenesis of these disorders (Caspani et al., 2019; Merchak and Gaultier, 2020). For instance, a recent study identified the molecule autoinducer-2 (AI-2) produced by segmented filamentous bacteria (SFB), a known inducer of gut Th17 cells (Ivanov et al., 2009), as one component of a cascade triggering depressive-like behavior in mice (Medina-Rodriguez et al., 2020). More specifically, AI-2 acts by increasing the levels of serum amyloid A 1 (SAA1) and SAA2 in the gut, which induce extensive Th17 differentiation. These cells then migrate to the hippocampus, causing depressive-like behavior in a CCL20/CCR6/IL-23-dependent fashion (Beurel et al., 2018). Although depressed patients were shown to have increased abundance of SFB (Medina-Rodriguez et al., 2020), this does not suffice to increase susceptibility to EAE (Regen et al., 2021). Furthermore, depressed patients had higher levels of fecal IL-17A, known to shift the gut microbiota and increase susceptibility to autoimmunity (Regen et al., 2021). These results highlight the complex interplay between the host, the gut microbiota, its metabolites and mood, although this still remain poorly understood in MS patients suffering from depression.

## Conclusion and Future Perspectives

**FIGURE 1:**
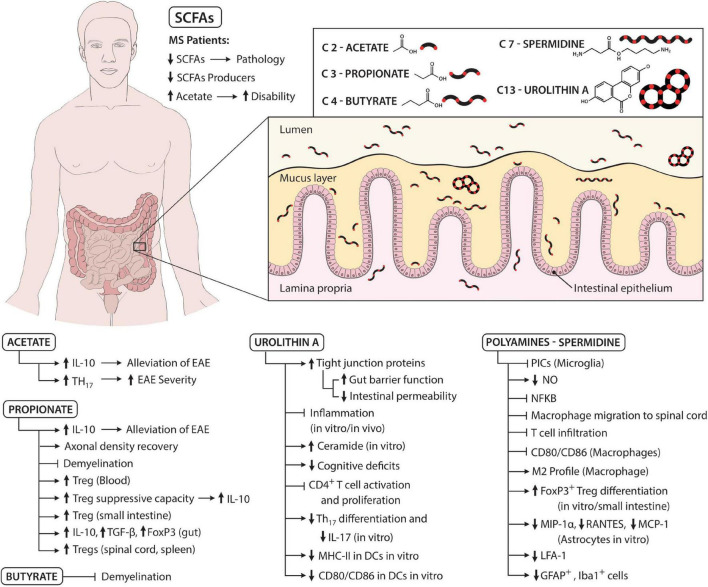
Schematic summarizing the role of microbial metabolites in the pathogenesis of MS. MS patients have reduced levels of SCFAs and SCFAs producers, which is associated with a more severe pathology. However, levels of acetate are increased in MS patients, which is linked with more disability. Acetate can both have detrimental and beneficial effects. For instance, acetate leads to more IL-10 secretion and thus alleviates EAE. On the other hand, acetate is also able to increase the frequency of Th17 cells and thus aggravate EAE. Propionate increases axonal density recovery and inhibits demyelination, the latter also being mediated by butyrate. Furthermore, propionate augments the frequency of Tregs (for instance, in the blood, spinal cord, and spleen) and their suppressive capacity while also upregulating IL-10, TGF-β, and FOXP3 levels in the gut. Polyamines, specifically spermidine, inhibit pro-inflammatory cytokines, and nitric oxide (NO) secreted by microglia likely *via* suppression of the NF-κB pathway. Moreover, spermidine inhibits macrophage and T cell migration to spinal cord and downregulates the levels of CD80 and CD86 on macrophages. Furthermore, spermidine shifts macrophages to the alternative or M2 profile, increases FOXP3^+^ Tregs differentiation, decreases chemokine secretion by astrocytes and the levels of LFA-1 on T cells and decreases the number of GFAP^+^ and Iba-1^+^ cells. Urolithins, specifically urolithin A (UA), increase tight junction protein levels and gut barrier function, which decreases intestinal permeability. Furthermore, UA inhibits inflammation, reduces cognitive deficits, diminishes Th17 differentiation, IL-17 secretion, MHC-II, CD80, and CD86 expression on DCs. Moreover, UA increases ceramide levels and blocks CD4^+^ T cell activation and proliferation. Altogether, these molecules, alone or synergistically, act to promote host homeostasis, but disease states and changes in diet can severely alter the gut microbiota composition and thus the gut microbial metabolites and favor disease initiation and progression.

Gut-derived metabolites play a significant, yet not fully understood, role in the development of MS. These molecules may act as guardians, preventing disease development *via* generation of Tregs and inhibition of a more pathogenic Th17 profile (Figure 1). However, their status can be drastically changed secondary to disease and diet, potentially causing a shift to a more pro-inflammatory response that favors disease progression. In this regard, direct supplementation with metabolites or a consumption of a diet rich in compounds known to promote Treg development and function may be of therapeutic benefit to MS patients, but further research is required to establish optimal therapeutic candidates and dosing regimen.

As many metabolites and the microorganisms that produce them are still undefined, future studies should focus on their identification and characterization, as this is likely to allow a direct modulation of disease *via* the gut and gut-brain axis, especially in terms of disease prevention. Furthermore, a more in-depth understanding of the contribution of the gut microbiota-derived metabolites in the relapse and remission as well as in the development of progressive form MS may also ensue after the establishment of this “metabolite library,” which may pave the way for a new era of therapeutics in MS.

## Author Contributions

ED-S conceived the study, performed the literature search, data collection, data analysis, and wrote the manuscript under the supervision of CP. CP and SM critically reviewed and edited the manuscript. All authors approved the final version of this manuscript.

## Conflict of Interest

The authors declare that the research was conducted in the absence of any commercial or financial relationships that could be construed as a potential conflict of interest.

## Publisher’s Note

All claims expressed in this article are solely those of the authors and do not necessarily represent those of their affiliated organizations, or those of the publisher, the editors and the reviewers. Any product that may be evaluated in this article, or claim that may be made by its manufacturer, is not guaranteed or endorsed by the publisher.
